# APOE-ε4 modulates the association among plasma Aβ_42_/Aβ_40_, vascular diseases, neurodegeneration and cognitive decline in non-demented elderly adults

**DOI:** 10.1038/s41398-022-01899-w

**Published:** 2022-03-29

**Authors:** Dai Shi, Siwei Xie, Anqi Li, Qingyong Wang, Hongbo Guo, Ying Han, Huaxi Xu, Wen-Biao Gan, Lei Zhang, Tengfei Guo, for the Alzheimer’s Disease Neuroimaging Initiative

**Affiliations:** 1 Department of Cerebrovascular Disease, the Fifth Affiliated Hospital, Sun Yat-sen University, Zhuhai, 519000 China; 2 Institute of Biomedical Engineering, Shenzhen Bay Laboratory, Shenzhen, 518132 China; 3 Department of Neurology, University of Chinese Academy of Sciences-Shenzhen Hospital, Shenzhen, 518107 China; 4 Department of Neurosurgery, Zhujiang Hospital, Southern Medical University, Guangzhou, 510282 China; 5 Department of Neurology, Xuanwu Hospital of Capital Medical University, Beijing, 100053 China; 6 Center for Brain Sciences, the First Affiliated Hospital of Xiamen University, Institute of Neuroscience, Fujian Provincial Key Laboratory of Neurodegenerative Disease and Aging Research, Xiamen University, Xiamen, 361000 China; 7 Institute of Neurological Diseases, Shenzhen Bay Laboratory, Shenzhen, 518132 China; 8 Institute of Biomedical Engineering, Peking University Shenzhen Graduate School, Shenzhen, 518055 China

**Keywords:** Predictive markers, Clinical genetics, Diagnostic markers

## Abstract

Including apolipoprotein E-ε4 (APOE-ε4) status and older age into consideration may increase the accuracy of plasma Aβ_42_/Aβ_40_ detecting Aβ+ individuals, but the rationale behind this remains to be fully understood. Besides, both Aβ pathology and vascular diseases are related to neurodegeneration and cognitive decline, but it is still not fully understood how APOE-ε4 modulates these relationships. In this study, we examined 241 non-demented Alzheimer’s Disease Neuroimaging Initiative participants to investigate the associations among age, white matter hyperintensities (WMH), hypertension, hyperlipidemia, body mass index (BMI), plasma Aβ_42_/Aβ_40_ measured by liquid chromatography tandem mass spectrometry, and ^18^F-florbetapir Aβ PET as well as their prediction of longitudinal adjusted hippocampal volume (aHCV) and cognition in APOE-ε4 carriers and non-carriers. We found older age predicted faster WMH increase (*p* = 0.024) and cortical Aβ accumulation (*p* = 0.043) in APOE-ε4 non-carriers only, whereas lower plasma Aβ_42_/Aβ_40_ predicted faster cortical Aβ accumulation (*p* < 0.018) regardless of APOE-ε4 status. While larger WMH and underweight predicted (*p* < 0.05) faster decreases in aHCV and cognition in APOE-ε4 non-carriers, lower plasma Aβ_42_/Aβ_40_ predicted (*p* < 0.031) faster decreases in aHCV and cognition in APOE-ε4 carriers. Higher Aβ PET also predicted faster rates of aHCV (*p* = 0.010) in APOE-ε4 carriers only, but was related to faster rates of cognitive decline (*p* < 0.022) regardless of APOE-ε4 status. These findings may provide novel insights into understanding different mechanisms underlie neurodegeneration and cognitive decline in non-demented elderly adults with and without APOE-ε4 allele, which may help the design of anti-Alzheimer’s clinical trials.

## Introduction

β-amyloid(Aβ) pathology of Alzheimer’s disease (AD) [1, 2] can be evaluated by either PET imaging [3–6] or cerebrospinal fluid (CSF) [7]. However, the highly-cost and limited-availability of PET imaging, and the side effect of invasive lumbar puncture limit their use in screening Aβ positive (Aβ+) individuals. Recent studies suggested that plasma Aβ_42_/Aβ_40_ measured by liquid chromatography tandem mass spectrometry (LC-MS/MS) [8–10] or enzyme-linked immunosorbent assay (ELISA) [11, 12] or ultrasensitive single molecule array (SIMOA) [13–15] techniques may be of advantage for screening individuals with high risk of AD [16]. Apolipoprotein E-ε4 (APOE-ε4) is the most important genetic risk factor of sporadic AD [17]. Combining older age, APOE-ε4 allele and plasma Aβ_42_/Aβ_40_ may increase the accuracy of detecting Aβ PET or CSF Aβ_42_/Aβ_40_ positive individuals or predicting future diagnosis [9–14], but the rationale behind this remains to be fully understood.

The Dominantly Inherited Alzheimer Network group [18] have suggested that white matter hyperintensities (WMH) may be closely linked to AD progression. A few studies [19–27] reported significant relationship between WMH and Aβ pathology, whereas other groups [28–33] observed opposite results. Besides, two studies [34, 35] demonstrated that APOE-ε4 carriers have higher WMH than APOE-ε4 non-carriers, whereas other studies [36–38] found that APOE-ε4 allele might be independent of cerebrovascular disease. Furthermore, both WMH [26, 39–43] and plasma Aβ_42_/Aβ_40_ [44–47] may be associated with neurodegeneration or cognitive decline. However, it remains unclear how APOE-ε4 modulates the relationships between Aβ and WMH as well as their association with neurodegeneration and cognitive decline. Rather than focus on the differences between APOE-ε4 carriers and non-carriers, we aim to determine the association among age, vascular diseases, plasma Aβ and cortical Aβ plaques in addition to how Aβ pathologies and vascular diseases predict longitudinal neurodegeneration and cognitive decline in APOE-ε4 carriers and non-carriers separately. We assume that Aβ pathologies and vascular diseases may play different roles in neurodegeneration and cognitive decline in APOE-ε4 carriers and non-carriers.

In this study, we examined non-demented Alzheimer’s Disease Neuroimaging Initiative (ADNI) participants to investigate the associations among age, vascular risk factors, WMH and plasma Aβ_42_/Aβ_40_, Aβ PET and their prediction of longitudinal WMH, cortical Aβ deposition, hippocampal atrophy, and cognitive decline in APOE-ε4 carriers and non-carriers. Our goal is to determine whether APOE-ε4 modulates the association between Aβ pathology and WMH, and their prediction of neurodegeneration and cognitive decline in non-demented elderly adults.

## Participants and methods

### Participants

Data used in this study were obtained from the ADNI database (ida.loni.usc.edu). The ADNI study was approved by institutional review boards of all participating centers, and written informed consent was obtained from all participants or their authorized representatives. In this study,126 cognitively unimpaired (CU) participants, and 115 participants with mild cognitive impairment (MCI) who had concurrent (acquisition intervals within 1 year) LC-MS/MS plasma Aβ_42_ and Aβ_40_, ^18^F-florbetapir (FBP) Aβ PET, structural MRI, WMH measurements, vascular risk factors, APOE-ε4 genotyping, and the cognitive test battery were included. Among 241 participants, 188 and 166 participants had at least 2-year’s longitudinal measurements of WMH and Aβ PET respectively, and 165 participants with at least two-year’s longitudinal MRI and cognitive tests measurements.

### Vascular risk factors

Body mass index (BMI) was calculated according to the formula: BMI = (body weight in kg)/(body height in meters [2]). Hyperlipidemia (HLD) (key words “hyperlipidemia” or “‘cholesterol”) and hypertension (HTN) (key words “hypertension” or “HTN” or “high blood pressure”) histories were defined as present or absent by searching text fields within the participants’ self-reported medical history (RECMHIST.csv and INITHEALTH.csv files downloaded from ADNI website at March 23, 2021).

### Plasma Aβ_42_ and Aβ_40_

LC-MS/MS plasma Aβ_40_ and Aβ_42_ were analyzed by the Washington University School of Medicine, St. Louis group. Briefly, targeted Aβ isoforms were immunoprecipitated with an anti-Aβ middomain antibody (HJ5.1) using a KingFisher (Thermo) automated immunoprecipitation platform. Immuno-enriched fractions were subsequently digested with Lys-N protease and subjected to LC-MS/MS as previously described [48] and also on the ADNI website (ida.loni.usc.edu). Absolute Aβ isoform concentrations were determined with a 15N-labeled internal standard for each isoform. The plasma Aβ_42_/Aβ_40_ ratio was calculated by dividing each plasma Aβ_42_ by plasma Aβ_40_.

### PET imaging and analysis

Details on FBP PET image acquisition and analysis are given elsewhere (http://adni-info.org). Briefly, PET data were acquired in five-min frames from 50–70 min post-injection (http://adni-info.org). Pre-processed FBP PET and structural MRI scans were downloaded from the LONI website (ida.loni.usc.edu). Cross-sectional (at the baseline timepoint) FBP standardized uptake value ratios (SUVRs) were calculated by dividing uptake across frontal, cingulate, parietal and temporal regions by that in the whole cerebellum to generate cortical summary COMPOSITE SUVRs [49]. Individuals with COMPOSITE FBP SUVR ≥1.11 were defined as Aβ+ as we described previously [7]. Considering that a composite reference region (made up of brainstem, whole cerebellum, and eroded white matter) [49] has shown superior stability in longitudinal analyses of Aβ PET, SUVRs that referred to the composite reference were used to investigate longitudinal changes of FBP SUVR.

### Hippocampal volume and white matter hyperintensities

Hippocampal volume (HCV) (cm^3^) was calculated across hemispheres from the structural MRI scans using Freesurfer, and adjusted by estimated total intracranial volume (TIV) using the approach employed by Jack et al. [50]. The adjusted hippocampal volume (aHCV) was calculated as the difference between the raw HCV and the expected HCV as described previously [51]. WMH was calculated at the University of California, Davis based on a Bayesian approach to segmentation of high resolution T1-weighted and FLAIR images as described previously [39] and also on the ADNI website. In order to compensate for individual variance in brain size and non-normal distribution, WMH was normalized to TIV and log_10_ transformed prior to analysis (Log_10_(WMH/TIV)).

### Preclinical Alzheimer cognitive composite scores

Preclinical Alzheimer’s Cognitive Composite (PACC) scores [52] were calculated by combing the standard z scores (using the mean values of all the ADNI CU participants) of the Delayed Recall portion of the Alzheimer’s Disease Assessment Scale, the delayed recall score on the logical memory IIa subtest from the Wechsler Memory Scale, the digit symbol substitution test score from the Wechsler Adult Intelligence Scale–Revised and the MMSE total score as we previously described [51].

### Statistical analysis

Normality of distributions was tested using the Shapiro-Wilk test and visual inspection of data. Data are presented as median (interquartile range (IQR)) or number (%) unless otherwise noted. Baseline characteristics were compared between APOE-ε4 carriers and non-carriers by using a two-tailed Mann-Whitney test or Fisher’s exact test.

In order to investigate how APOE-ε4 status affects the associations among age, vascular disease risk factors, WMH, and plasma Aβ_42_/Aβ_40_, we used generalized linear model (GLM) to examine the relationships of WMH and plasma Aβ_42_/Aβ_40_ with age, sex, HTN, HLD and BMI in APOE-ε4 non-carriers and carriers, adjusting for the diagnosis status. Afterwards, we first studied the associations of Aβ PET with age, plasma Aβ_42_/Aβ_40_ and WMH using Pearson’s correlation test, and further used GLM models to determine the cross-sectional relation of Aβ PET with plasma Aβ_42_/Aβ_40_ and WMH in APOE-ε4 non-carriers and carriers, adjusting for age, sex, and diagnosis status.

Subsequently, we used linear mixed-effect (LME) models to investigate the prediction of longitudinal WMH changes over time by baseline plasma Aβ_42_/Aβ_40_ and Aβ PET, and the prediction of longitudinal Aβ PET changes over time by baseline plasma Aβ_42_/Aβ_40_ and WMH in APOE-ε4 non-carriers and carriers, adjusting for age, sex, HTN, HLD, and BMI as well as their interaction with time, diagnosis status, and including a random slope and intercept for each participant.

In order to investigate how APOE-ε4 status affects the predictive effects of baseline plasma Aβ_42_/Aβ_40_, Aβ PET and WMH on perspective neurodegeneration and cognitive decline, we used LME models to study how baseline plasma Aβ_42_/Aβ_40_, Aβ PET and WMH predict longitudinal changes of aHCV and PACC over time in APOE-ε4 non-carriers and carriers, including the interaction of HTN and time, HLD and time, BMI and time, and adjusting for age, sex, education and diagnosis status.

Finally, we used LME models (including a random slope and intercept for each participant) to estimate: (1) annual rate of aHCV (ΔaHCV), adjusting for sex and diagnosis; (2) annual rate of PACC change (ΔPACC), adjusting for sex, education and diagnosis. Considering that WMH and plasma Aβ_42_/Aβ_40_ were related to ΔaHCV and ΔPACC in APOE-ε4 non-carriers and APOE-ε4 carriers respectively (See Figs. 3, 4 in Results), we then conducted the mediation analyses among age, WMH and ΔaHCV, and among WMH, ΔaHCV and ΔPACC in APOE-ε4 non-carriers, and the mediation analyses among plasma Aβ_42_/Aβ_40_, Aβ PET and ΔaHCV, and among plasma Aβ_42_/Aβ_40_, ΔaHCV and ΔPACC in APOE-ε4 carriers using latent variable modeling [53] (R; Lavaan package).

We selected two-sided *p* < 0.05 as the significance level unless otherwise noted. In the mediation analyses, all the variables were converted to standard z scores. Total, direct, and indirect associations were calculated via a 5000-iteration bootstrapping procedure. Longitudinal data of biomarkers were defined as the data that was closest in time to, and after, the baseline plasma Aβ_42_/Aβ_40_. Statistical analyses were performed in the statistical program *R* (v4.0.2, The R Foundation for Statistical Computing) unless otherwise noted.

## Results

### Demographics

Data in this study were acquired in ADNI between July 2010 and March 2021. The characteristics of 241 participants analyzed in this study can be found in Table 1. In total, 92 (38.2%) individuals were APOE-ε4 carriers. At baseline, APOE-ε4 carriers had slightly younger age, lower plasma Aβ_42_/Aβ_40_, higher FBP SUVR, and higher percentages of Aβ PET positivity than APOE-ε4 non-carriers, while no other difference was found. Notably, the percentage of MCI individuals between APOE-ε4 carriers and non-carriers was not significantly different from each other. Longitudinal data of different biomarkers were shown in Table 1 as well.

**Table 1:** Demographics of participants in this study.

	APOE-ε4 non-carriers	APOE-ε4 carriers
Sample size	149	92
MCI (No., %))	66 (44.3%)	49 (53.3%)
Age (median (IQR))	74.3 (9.0)	73.0 (11.7)*
Education (median (IQR))	17 (4)	16 (4.25)
Females (*N*, %)	79 (47.0%)	42 (54.3%)
WMH (median (IQR))	−2.57 (0.64)	−2.70 (0.76)
Hyperlipidemia (No., %)	55 (36.9%)	33 (35.9%)
Hypertension (No., %)	56 (37.6%)	28 (30.4%)
BMI (Median (IQR))	27.0 (5.2)	25.9 (6.5)
Plasma Aβ_42_/Aβ_40_ (Median (IQR))	0.1208 (0.0159)	0.1152 (0.0157)***
FBP SUVR (Median (IQR))	1.04 (0.18)	1.21 (0.32)****
Aβ PET positivity (No., %)	50 (33.6%)	60 (65.2%)*****
aHCV (Median (IQR))	−0.18 (1.55)	−0.13 (1.35)
PACC (Median (IQR))	−1.23 (4.91)	−1.62 (5.90)
Longitudinal WMH (*n* = 188, duration of years: 4.3 (4.4, 2.0–9.4), scans: 6 (3.25, 2–10))		
Sample size	118	70
Duration, year (Median (IQR, range))	4.6 (4.6, 2.0–9.4)	4.3 (4.3, 2.0–8.6)
No. of visits (Median (IQR, range))	6 (4, 2–10)	6 (3, 2–10)
Longitudinal Aβ PET (*n* = 166, duration of years: 5.6 (4.0, 2.0–9.2), scans: 3 (2, 2–5))		
Sample size	105	61
Duration, year (Median (IQR, range))	5.8 (4.0, 2.0–9.2)	5.5 (3.9, 2.0–8.7)
No. of visits (Median (IQR, range))	3 (2, 2–5)	3 (2, 2–5)
Longitudinal aHCV (*n* = 165, duration of years: 5.6 (4.2, 2.0–9.1), scans: 6 (3, 2–10))		
Sample size	103	62
Duration, year (Median (IQR, range))	5.9 (4.2, 2.0–9.1)	5.1 (4.2, 2.0–8.6)
No. of visits (Median (IQR, range))	6 (3.5, 2–10)	6 (2.75, 2–10)
Longitudinal PACC (*n* = 165, duration of years: 6.0 (3.6, 2.0–9.4), visits: 6 (3, 2–11))		
Sample size	103	62
Duration, year (Median (IQR, range))	6.0 (3.7, 2.0–9.4)	5.8 (4.1, 2.0–9.1)
No. of visits (Median (IQR, range))	6 (3, 2–10)	6 (4, 2–11)

*Aβ* Amyloid-β, *aHCV* Adjusted hippocampal volume, *BMI* Body mass index, *FBP* 18F-florbetapir, *FDG* 18F-fluorodeoxyglucose, *IQR* Interquartile range, *MCI* Mild cognitive impairment, *PACC* Preclinical Alzheimer Cognitive Composite, *SUVR* Standardized uptake value ratio, *WMH* White matter hyperintensities.

*p = 0.015; **p < 0.001, ***p < 0.001, ****p < 0.001, Mann–Whitney *U* test; *****p < 0.001, Fisher’s exact test.

### The cross-sectional association among age, vascular risk disease, plasma Aβ_42_/Aβ_40_, and Aβ PET

Greater WMH was related to older age in both APOE-ε4 non-carriers (standardized β value (β_std_) = 0.46 [95% ci, 0.28, 0.64], *p* < 0.001) and APOE-ε4 carriers (β_std_ = 0.42 [95% ci, 0.18, 0.66], *p* = 0.001), and was related to HTN (β_std_ = 0.53 [95% ci, 0.13, 0.92], *p* = 0.010) in APOE-ε4 non-carriers only (Supplemental Fig. 1A, B). No significant association was found among age, vascular risk factors and plasma Aβ_42_/Aβ_40_ (Supplemental Fig. 1C, D). Older age, lower plasma Aβ_42_/Aβ_40_ and greater WMH were significantly associated with higher FBP SUVR regardless of APOE-ε4 status (Supplemental Fig. 2), whereas lower plasma Aβ_42_/Aβ_40_ but not greater WMH was significantly related to higher FBP SUVR after adjusting for age and sex (Supplemental Fig. 3).

### Prediction of longitudinal WMH and Aβ PET

**Fig. 1:**
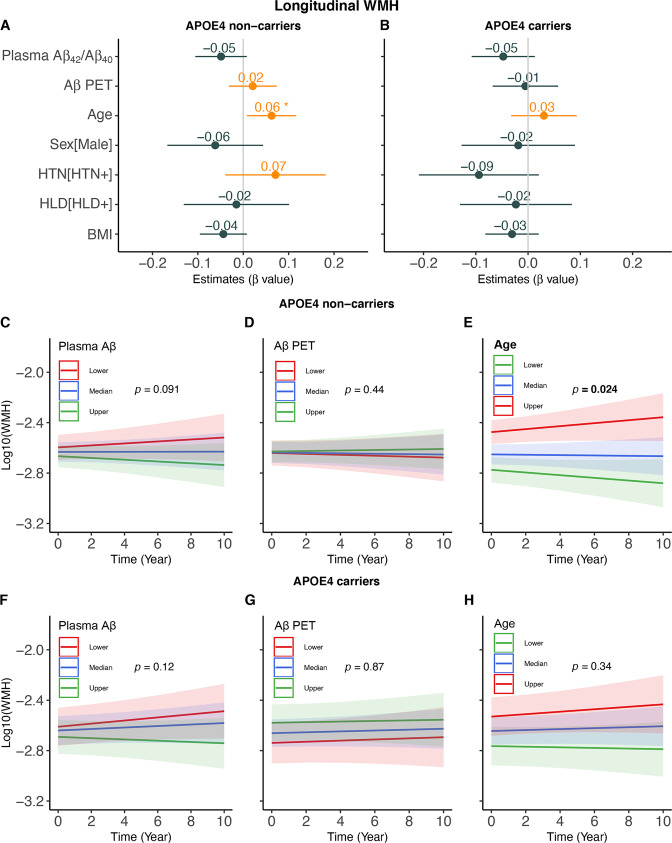
Predictors of longitudinal WMH changes. The estimated β values of each predictor for longitudinal WMH changes in linear mixed effect models in (**A**) APOE-ε4 non-carriers and (**B**) APOE-ε4 carriers. Prediction of longitudinal WMH changes over time by baseline plasma Aβ_42_/Aβ_40_, Aβ PET and age in APOE-ε4 non-carriers (**C**–**E**) and carriers (**F**–**H**). Note: lower, median and upper represent the value that cuts off the first 25%, 50% and 75% of the data when it is sorted in ascending order.

**Fig. 2:**
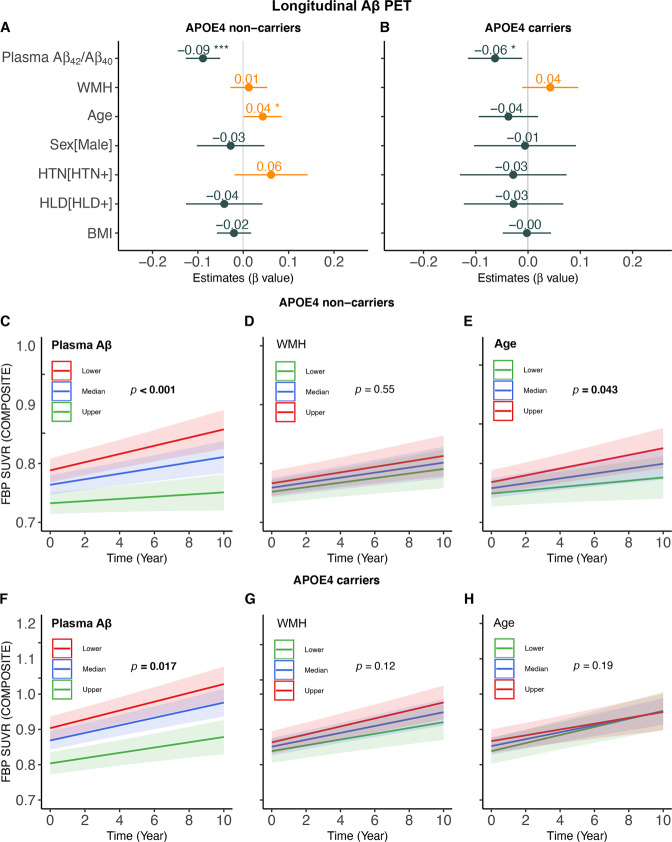
Predictors of longitudinal Aβ PET changes. The estimated β values of each predictor for longitudinal Aβ PET changes in linear mixed effect models in (**A**) APOE-ε4 non-carriers and (**B**) APOE-ε4 carriers. Prediction of longitudinal Aβ PET changes over time by baseline plasma Aβ_42_/Aβ_40_, WMH and age in APOE-ε4 non-carriers (**C**–**E**) and APOE-ε4 carriers (**F**–**H**). Note: lower, median and upper represent the value that cuts off the first 25%, 50% and 75% of the data when it is sorted in ascending order.

At follow-up, older age was associated with faster WMH increase over time in APOE-ε4 non-carriers (β_std_ = 0.0625 [95% CI, 0.0083, 0.1167], *p* = 0.024) but not in APOE-ε4 carriers (Fig. 1A, B, E, H). No other significant predictor of longitudinal WMH changes was found regardless of APOE-ε4 status (Fig. 1). Lower plasma Aβ_42_/Aβ_40_ but not greater WMH at baseline was related to faster rates of FBP SUVR increase in both APOE-ε4 non-carriers (β_std_ = −0.0887 [95% CI, −0.1265, −0.0509], *p* < 0.001) and APOE-ε4 carriers (β_std_ = −0.0631 [95% CI, −0.1152, −0.0111], *p* = 0.017) (Fig. 2A–C, F). Older age also predicted faster increases in FBP SUVR (β_std_ = 0.0430 [95% CI, 0.0013, 0.0847], *p* = 0.043) in APOE-ε4 non-carriers but not in APOE-ε4 carriers (Fig. 2E, H).

### Prediction of longitudinal hippocampal atrophy and cognitive decline

**Fig. 3:**
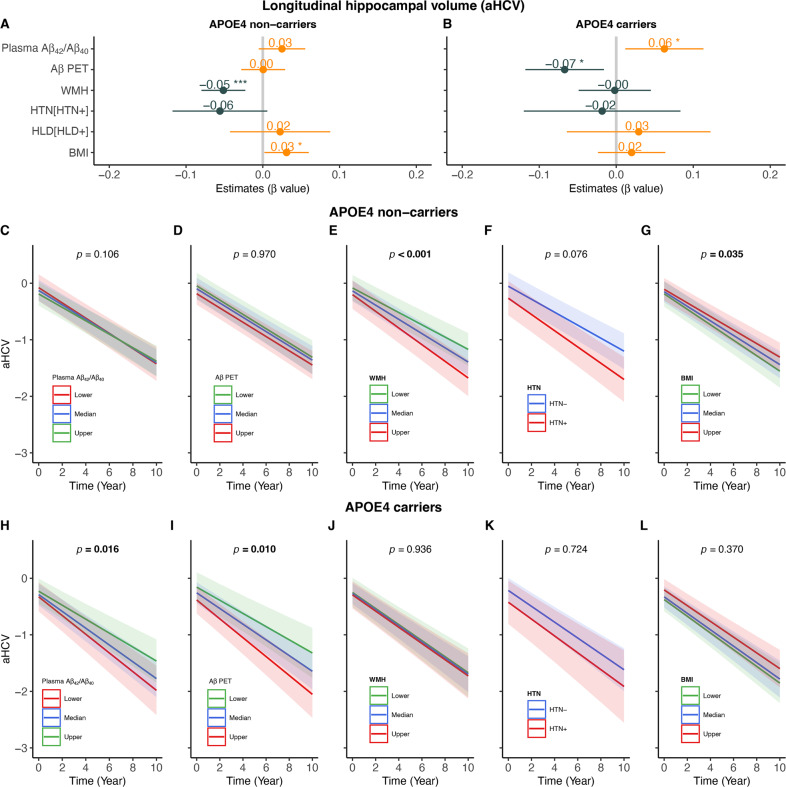
Predictors of longitudinal hippocampal atrophy. Associations of longitudinal adjusted hippocampal volume (aHCV) with baseline plasma Aβ_42_/Aβ_40_, Aβ PET, WMH, hypertension (HTN), hyperlipidemia (HLD) and body mass index (BMI) in (**A**) APOE-ε4 non-carriers and (**B**) APOE-ε4 non-carriers. Prediction of longitudinal aHCV changes over time by baseline plasma Aβ_42_/Aβ_40_, Aβ PET, WMH, HTN and BMI in APOE-ε4 non-carriers (**C**–**G**) and APOE-ε4 carriers (**H**–**L**). Notes: APOE-ε4 non-carriers (HTN−: *n* = 60, HTN+: *n* = 43), APOE-ε4 carriers (HTN−: *n* = 44, HTN+: *n* = 18); lower, median and upper represent the value that cuts off the first 25%, 50% and 75% of the data when it is sorted in ascending order.

The predictors of longitudinal aHCV changes over time in APOE-ε4 non-carriers and carriers were summarized in Fig. 3A, B. In APOE-ε4 non-carriers, greater WMH (Fig. 3E, β_std_ = −0.051 [95% CI, −0.080, −0.023], *p* < 0.001) but not lower plasma Aβ_42_/Aβ_40_ (Fig. 3C) and higher FBP SUVR (Fig. 3D) at baseline predicted faster decreases in aHCV. Lower BMI (Fig. 3G, β_std_ = 0.031 [95% CI, 0.002, 0.060], *p* = 0.035) was also associated with faster rates of aHCV decreases. In contrast, lower plasma Aβ_42_/Aβ_40_ (Fig. 3H, β_std_ = 0.063 [95% CI, 0.012, 0.113], *p* = 0.016) and higher FBP SUVR (Fig. 3I, β_std_ = −0.067 [95% CI, −0.118, −0.016], *p* = 0.010) but not greater WMH (Fig. 3J) and lower BMI (Fig. 3L) at baseline predicted faster rates of aHCV decreases in APOE-ε4 carriers. No other significant predictor was found.

**Fig. 4:**
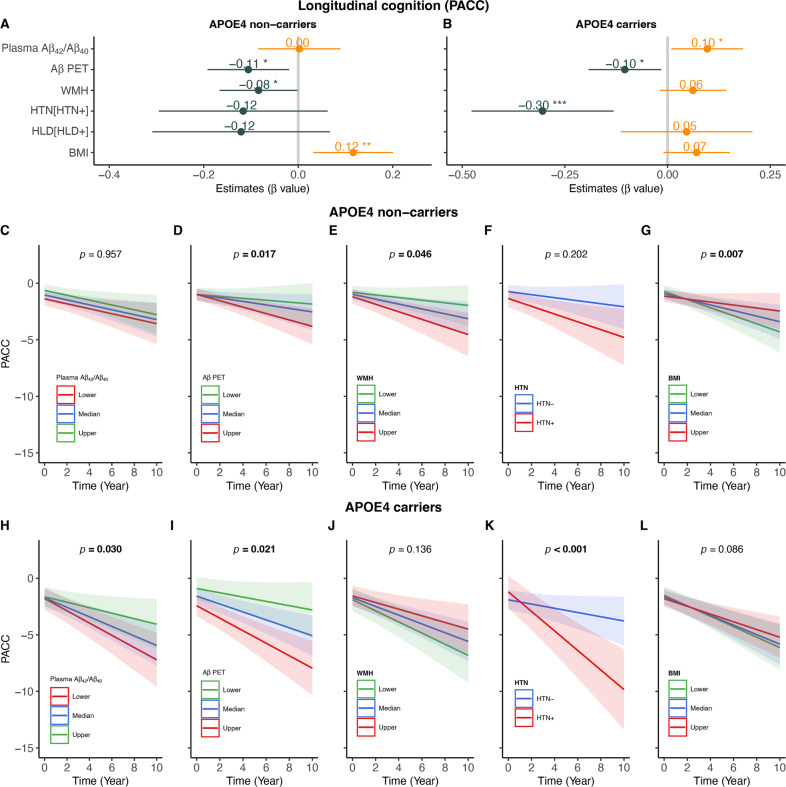
Predictors of longitudinal cognitive decline. Associations of longitudinal PACC changes over time by baseline plasma Aβ_42_/Aβ_40_, Aβ PET, WMH, hypertension (HTN), Hyperlipidemia (HLD) and body mass index (BMI) in (**A**) APOE-ε4 non-carriers and (**B**) APOE-ε4 non-carriers. Prediction of longitudinal PACC changes over time by baseline plasma Aβ_42_/Aβ_40_, Aβ PET (FBP SUVR), WMH, HTN and BMI in APOE-ε4 non-carriers (**C**–**G**) and carriers (**H**–**L**). Notes: APOE-ε4 non-carriers (HTN−: *n* = 60, HTN+: *n* = 43), APOE-ε4 carriers (HTN−: *n* = 44, HTN+: *n* = 18); lower, median and upper represent the value that cuts off the first 25%, 50% and 75% of the data when it is sorted in ascending order.

The predictors of longitudinal PACC changes over time in APOE-ε4 non-carriers and carriers were summarized in Fig. 4A, B. In APOE-ε4 non-carriers, higher FBP SUVR (Fig. 4D, β_std_ = −0.106 [95% CI, −0.192, −0.019], *p* = 0.017), greater WMH (Fig. 4E, β_std_ = −0.084 [95% CI, −0.167, −0.002], *p* = 0.046) and lower BMI (Fig. 4G, β_std_ = 0.116 [95% CI, 0.032, 0.201], *p* = 0.007) but not lower plasma Aβ_42_/Aβ_40_ (Fig. 4C) at baseline predicted faster PACC decline. In contrast, lower plasma Aβ_42_/Aβ_40_ (Fig. 4H, β_std_ = 0.097[95% CI, 0.009, 0.184], *p* = 0.030), higher FBP SUVR (Fig. 4I, β_std_ = −0.104 [95% CI, −0.193, −0.016], *p* = 0.021) and HTN (Fig. 4K, β_std_ = −0.304 [95% CI, −0.477, −0.132], *p* < 0.001) but not greater WMH (Fig. 4J) and lower BMI (Fig. 4L) at baseline predicted faster PACC decline (Fig.4F, G, I) in APOE-ε4 carriers.

### The mediation analyses among age, WMH, Aβ pathology, neurodegeneration and cognitive decline

In the mediation analyses among age, WMH, ΔaHCV, and ΔPACC in APOE-ε4 non-carriers, WMH explained 24.4% (−0.11/(−0.45) = 0.244) of the association between age and ΔaHCV (Supplemental Fig. 4A), which explained 46.9% (−0.15/(−0.32) = 0.469) of the association between WMH and ΔPACC in APOE-ε4 non-carriers (Supplemental Fig. 4B).

In the mediation analyses among plasma Aβ_42_/Aβ_40_, Aβ PET, ΔaHCV, and ΔPACC in APOE-ε4 carriers, Aβ PET explained 31.5% (0.17/0.54 = 0.315) of the association between plasma Aβ_42_/Aβ_40_ and ΔaHCV (Supplemental Fig. 4C), which explained 74.4% (0.32/0.43 = 0.744) of the association between plasma Aβ_42_/Aβ_40_ and ΔPACC in APOE-ε4 carriers (Supplemental Fig. 4D).

## Discussion

In this study, we investigated the relationships among age, vascular risk diseases, plasma Aβ_42_/Aβ_40_, Aβ PET, neurodegeneration and cognitive decline in non-demented elderly adults with and without APOE-ε4 allele respectively. Older age predicted faster rates of WMH increase and Aβ accumulation in APOE-ε4 non-carriers only, whereas lower plasma Aβ_42_/Aβ_40_ but not greater WMH predicted faster rates of Aβ accumulation regardless of APOE-ε4 status. Importantly, we found lower plasma Aβ_42_/Aβ_40_ predicted faster rates of hippocampal atrophy and cognitive decline in APOE-ε4 carriers only independent of cortical Aβ burden. Higher Aβ PET also predicted faster hippocampal atrophy over time in APOE-ε4 carriers only, but was related to faster cognitive decline regardless of APOE-ε4 status. In contrast, greater WMH and lower BMI predicted faster hippocampal atrophy and cognitive decline in APOE-ε4 non-carriers, implying vascular risk factors play an important role in neurodegeneration and cognitive decline in non-demented elderly adults without APOE-ε4 allele. These findings support our hypothesis that Aβ pathologies and vascular diseases play distinct roles in hippocampal atrophy and cognitive decline in non-demented elderly adults with and without APOE-ε4 allele.

Consistent with a few recent literatures [9–14], we found APOE-ε4 carriers showed lower plasma Aβ_42_/Aβ_40_, higher cortical Aβ deposition and larger probability of Aβ PET positivity compared to APOE-ε4 non-carriers. One genome-wide association study [54] found that APOE-ε4 allele had the strongest association with Aβ_42_ levels but not with Aβ_40_ levels in plasma measured by enzyme-linked immunosorbent assay (ELISA), and was significantly related to lower plasma Aβ_42_/Aβ_40_. However, we did not find significant relation among age, vascular risk factors and plasma Aβ_42_/Aβ_40_ regardless of APOE-ε4 status, which was in agreement with one previous ADNI study [28] in which plasma Aβ was measured by ELISA approach without considering APOE-ε4 status. Longitudinally, we found lower plasma Aβ_42_/Aβ_40_ predicted faster rates of Aβ accumulation regardless of APOE-ε4 status, which was consistent with the recent findings reported by the BIOFINER group [47]. In addition, we also found older age was related to faster Aβ accumulation rates in the absence of APOE-ε4 allele, providing further evidence for explaining why combing lower plasma Aβ_42_/Aβ_40_ and older age can improve the accuracy of detecting amyloid positivity defined by CSF [11, 14] or PET [9].

Previous studies [44–47] have reported significant association between plasma Aβ_42_/Aβ_40_ and neurodegeneration or cognitive decline, although none of them investigated how APOE-ε4 affects these relationships. Importantly, we further found that lower plasma Aβ_42_/Aβ_40_ predicted longitudinal neurodegeneration and cognitive decline in APOE-ε4 carriers only, but did not show significant predictive effect in APOE-ε4 non-carriers over around 5–6 years of median follow-up. These findings indicate that plasma Aβ_42_/Aβ_40_ may be useful for screening APOE-ε4 carriers (such as Alzheimer’s Prevention Initiative (API) Generation Study) as the potential participants for anti-AD clinical trials with neurodegeneration or cognitive decline as the ending points, whereas its application may be limited in non-demented elderly adults without APOE-ε4 allele.

Furthermore, we noticed that plasma Aβ_42_/Aβ_40_ and cortical Aβ burden independently predicted longitudinal hippocampal atrophy in APOE-ε4 carriers but not in APOE-ε4 non-carriers, suggesting that APOE-ε4 allele may probably modulate the association between Aβ pathology and hippocampal atrophy. The mediation analyses provided further evidence that cortical Aβ burden only partially explained the association between plasma Aβ_42_/Aβ_40_ and longitudinal hippocampal atrophy, which fully mediated the association between plasma Aβ_42_/Aβ_40_ and cognitive decline in APOE-ε4 carriers. In contrast, we found elevated cortical Aβ deposition significantly predicted longitudinal cognitive decline regardless of APOE-ε4 status, which may be explained by that increased cortical Aβ burden may be related to other aspect of neurodegeneration [55] that resulting in cognitive decline in addition to hippocampal atrophy in APOE-ε4 non-carriers. Together, these findings suggest that plasma Aβ_42_/Aβ_40_ may only detect one aspect of Aβ pathology even in the presence of APOE-ε4 allele, but lower plasma Aβ_42_/Aβ_40_ may be related to hippocampal atrophy independent of cortical Aβ burden in APOE-ε4 carriers.

In line with one recent study [33], we found greater WMH was related to higher cortical Aβ burden in univariate regression analyses, but this association disappeared after adjusting for age. Unlike a few reports [21, 23, 26], we found greater baseline WMH did not predict longitudinal cortical Aβ accumulation, neither lower baseline plasma Aβ_42_/Aβ_40_ nor higher cortical Aβ deposition predicted longitudinal WMH increase regardless of APOE-ε4 status. Consistent with our findings, several cross-sectional studies [28–33] did not find relation between WMH and Aβ pathology measured by CSF, PET imaging or immunohistochemistry. The distinct clinical status of participants may be related with the discrepancy, because previous studies reported no significant association between Aβ pathology and WMH in non-demented elderly adults [29, 32] but did find they related to each other in cohorts with demented elderly adults [26, 32]. Besides, the discordance may be also due to the fact that we did the analyses in APOE-ε4 non-carriers and carriers separately.

Older age showed strong correlation with greater WMH at baseline and predicted longitudinal WMH increases in APOE-ε4 non-carriers, suggesting older age may be tightly linked to white matter lesions in the absence of APOE-ε4 allele. Another key finding of this study was that age-related WMH increase predicted longitudinal hippocampal atrophy and cognitive decline in APOE-ε4 non-carriers. Consistent with our findings, the Mayo clinic group [43] and one previous ADNI study [39] also found older age was one significant predictor of vascular health, which showed direct correlation with AD pattern neurodegeneration or cognitive decline. One recent study [26] found APOE-ε4 only affected the associations of Aβ pathology with neurodegeneration and cognition, whereas did not modulate the associations of WMH with longitudinal neurodegeneration and cognitive decline. This suggests that greater WMH may affect brain atrophy and cognitive decline independent of APOE-ε4 allele. The mediation analyses between WMH-related neurodegeneration and cognitive decline in APOE-ε4 non-carriers showed that greater WMH partially explained the age-related hippocampal atrophy, which mediated the association between WMH and cognitive decline in APOE-ε4 non-carriers. In accordance with the findings of one previous study [56] that being underweight could increase the risk of dementia, we further demonstrated lower BMI predicted faster rates of hippocampal atrophy and cognitive decline in APOE-ε4 non-carriers, implying underweight may be also a risk factor of AD in non-demented elder adults without APOE-ε4 allele. Altogether, it is likely that older age-associated vascular diseases and underweight may contribute to faster neurodegeneration and cognitive decline in elderly adults without APOE-ε4 allele.

The strength of this study is that we analyzed how APOE-ε4 allele modulates the cross-sectional and longitudinal associations between plasma Aβ_42_/Aβ_40_, Aβ PET and white matter lesions as well as their prediction of longitudinal hippocampal atrophy and cognitive decline simultaneously up to 9 years’ follow-up. However, this study also has limitations. First, the inclusion criteria of ADNI excluded subjects with important vascular pathology, thus our analyses on vascular risk factors should be taken cautiously. Second, ADNI did not have longitudinal LC-MS/MS plasma Aβ_42_/Aβ_40_ data at this moment, so further longitudinal data and other techniques (ELISA [11, 12] or ultrasensitive single molecule array [13–15]) would be useful to validate our findings. Third, we noticed that APOE-ε4 carriers with hypertension showed faster rates of cognitive decline than those without hypertension, but the interpretation of this findings may be limited due to the relatively small sample size (*n* = 18) of APOE-ε4 carriers with hypertension. Considering that the academic community becomes increasingly concerned about the overuse and misinterpretation of significance testing and p values [57], thus the 95% ci values may be more informative than their corresponding p values in our statistical analysis.

In conclusions, this study suggests that lower plasma Aβ_42_/Aβ_40_ may predict hippocampal atrophy and cognitive decline in APOE-ε4 carriers, whereas the white matter lesion and underweight are more involved in hippocampal atrophy and cognitive decline of APOE-ε4 non-carriers. These findings are important for understanding different mechanisms related to neurodegeneration and cognitive decline in APOE-ε4 carriers and non-carriers, providing significant reference for anti-AD clinical trials.

## Supplementary information

Supplementary Material
