# Supplementary material for: APOE-ε4 modulates the association among plasma Aβ42/Aβ40, vascular diseases, neurodegeneration and cognitive decline in non-demented elderly adults
Source: Transl Psychiatry. 2022 Mar 29;12:128. doi: 10.1038/s41398-022-01899-w (PMC8964707; doi:10.1038/s41398-022-01899-w)
Supplement: Supplementary file 1 — Supplementary Material [file 41398_2022_1899_MOESM1_ESM.pdf]

Supplemental Material

The association of age, vascular risk disease, plasma Aβ$_{42}$/Aβ$_{40}$, and Aβ PET

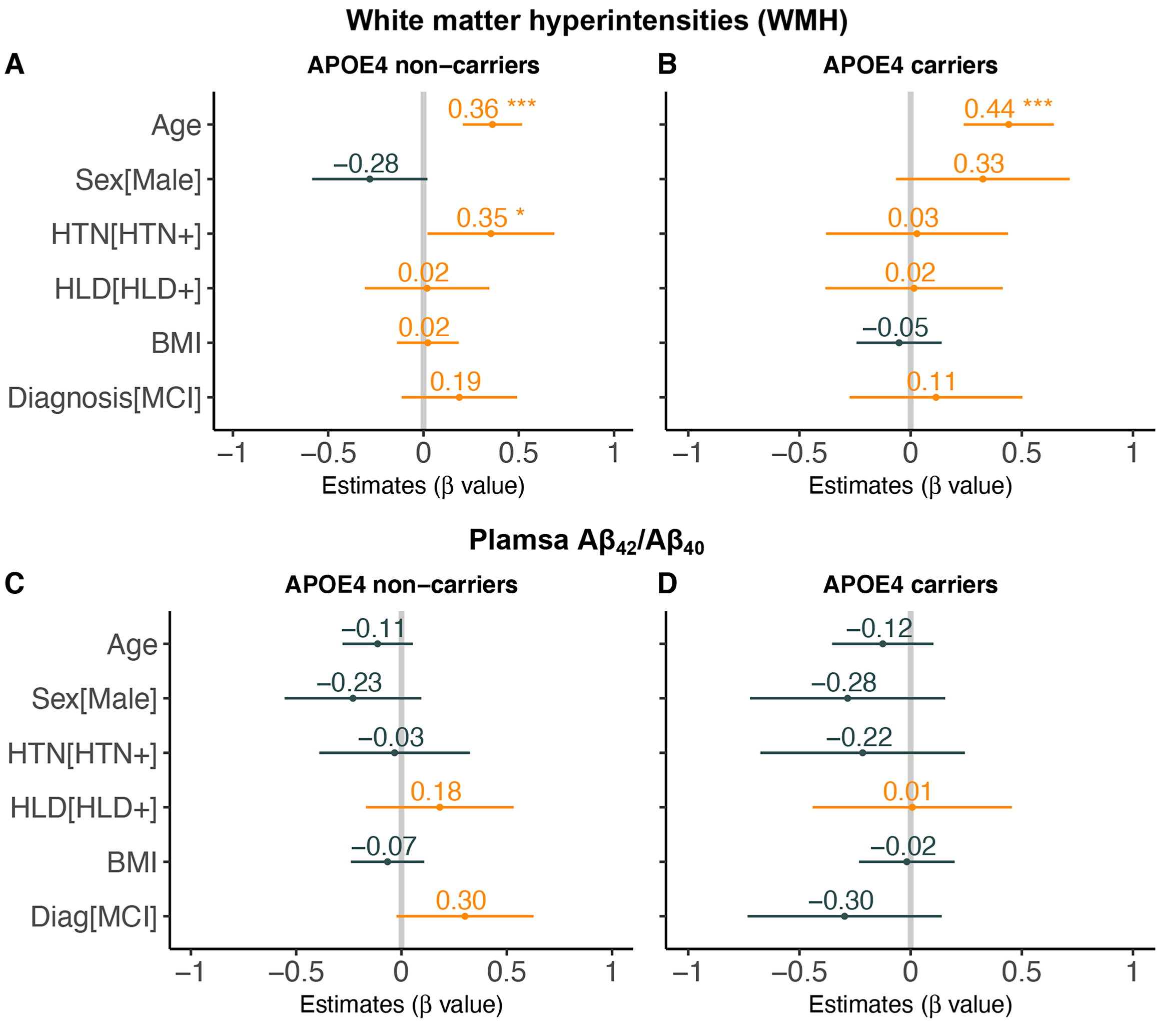

Supplemental fig. 1. Risk factors related to white matter intensities and plasma Aβ$_{42}$/Aβ$_{40}$ in APOE-ε4 carriers and non-carriers. *The associations of age and vascular risk factors with white matter intensities (WMH) (A and B) and plasma Aβ$_{42}$/Aβ$_{40}$ (C and D) in (A)APOE-ε4 non-carriers and (B) carriers.*

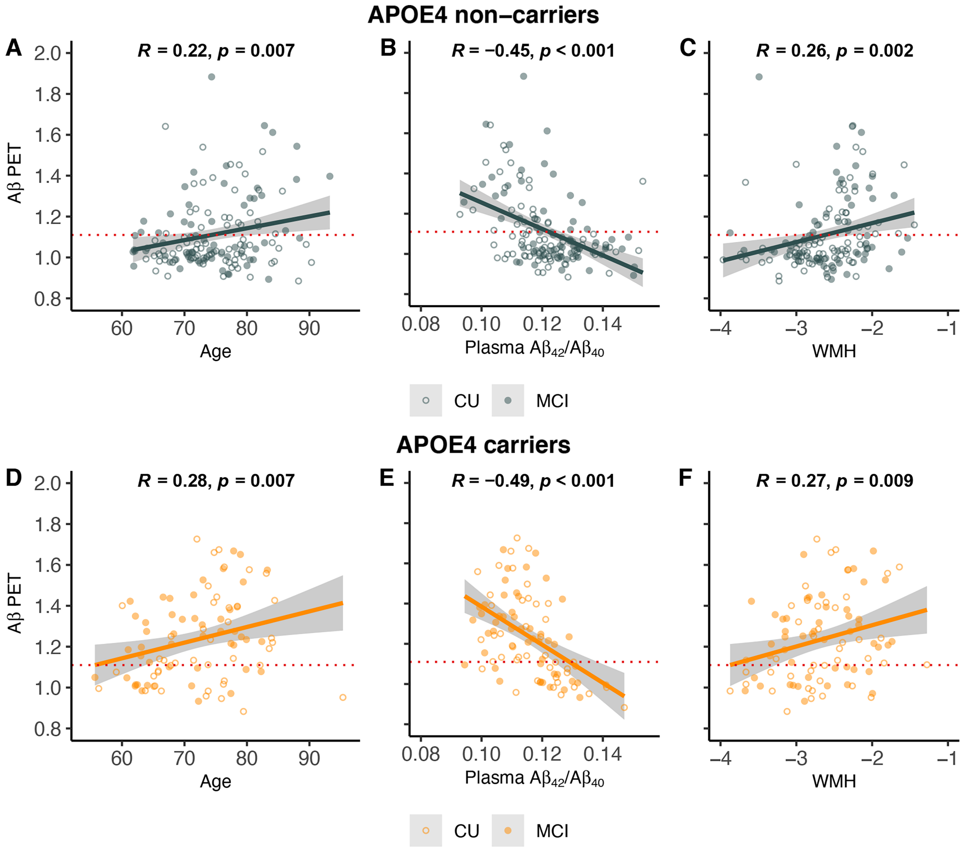

Supplemental fig. 2. *The association among age, WMH, plasma $A\beta_{42}/A\beta_{40}$, and Aβ PET in (A-C) APOE-ε4 non-carriers and (B)carriers.*

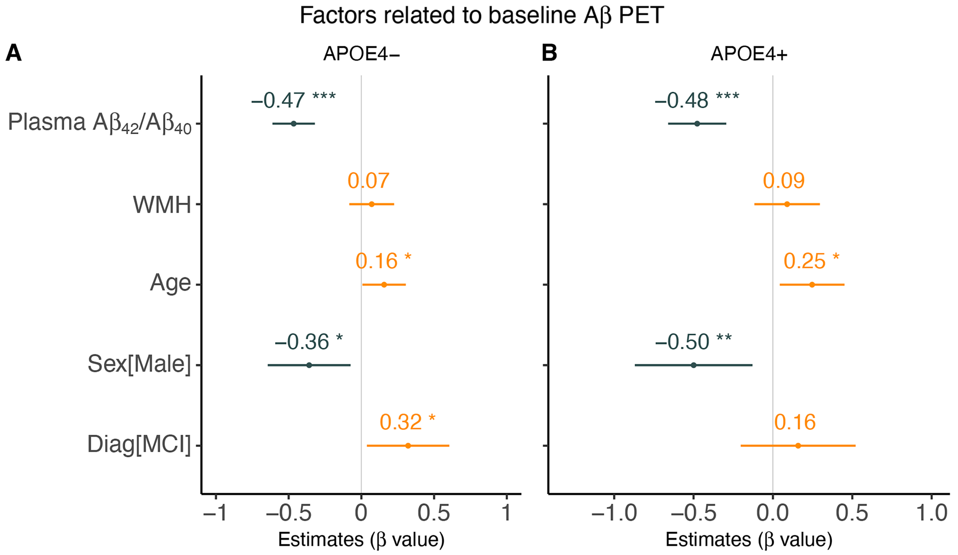

Supplemental fig. 3. *Risk factors related to baseline Aβ PET in (A) APOE-ε4 non-carriers and (B) carriers.*

The mediation analyses among age, WMH, Aβ pathology, neurodegeneration and cognitive decline

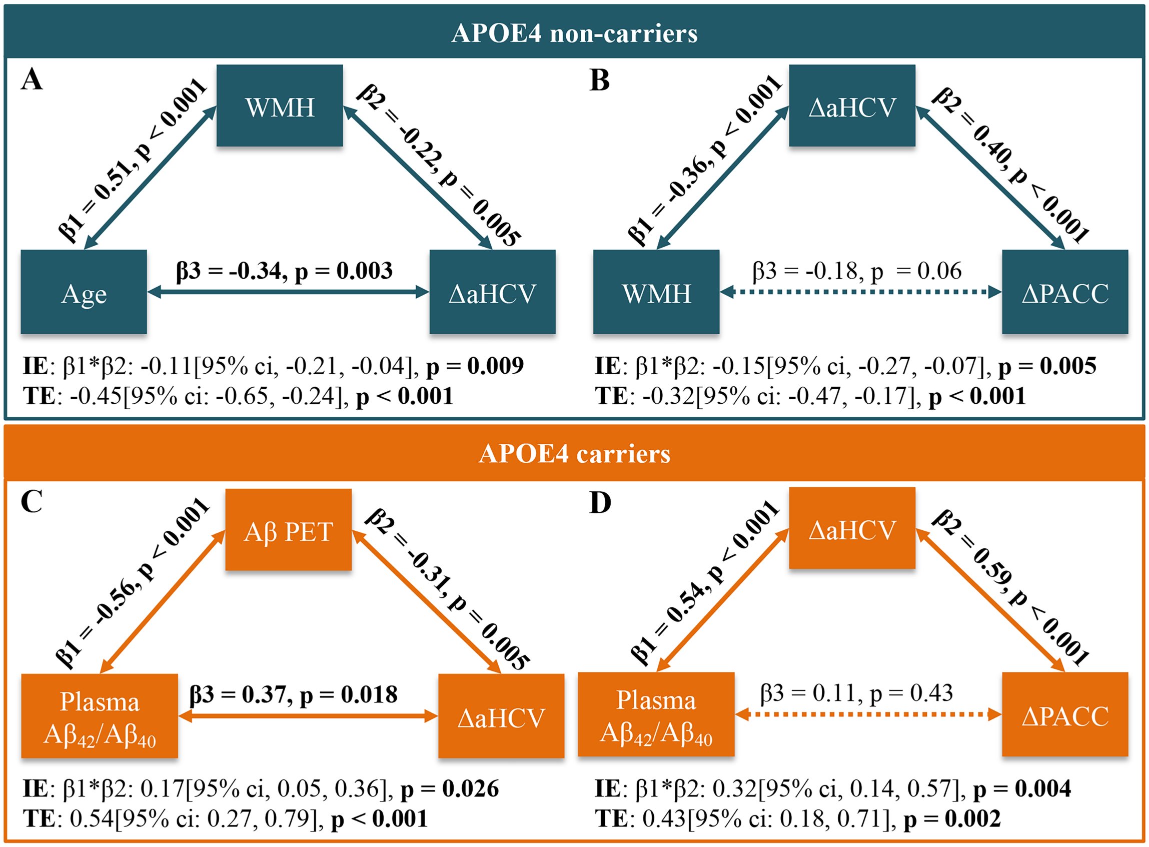

Supplemental fig. 4. Sequential mediation analyses between age, WMH, plasma $A\beta_{42}/A\beta_{40}$, Aβ PET, hippocampal atrophy and cognitive decline in APOE-ε4 non-carriers and carriers. *(A) WMH partially explained the association between age and slope of aHCV (ΔaHCV), and (B) ΔaHCV mediated the association between WMH and slope of PACC (ΔPACC) in APOE-ε4 non-carriers. (C) Aβ PET partially explained the association between plasma $A\beta_{42}/A\beta_{40}$ and slope of ΔaHCV, and (D) ΔaHCV mediated the association between plasma $A\beta_{42}/A\beta_{40}$ and ΔPACC in APOE-ε4 carriers.*
